# Advancements in Mass Spectrometry‐Based Glycomics in Food and Nutritional Science

**DOI:** 10.1002/mas.70003

**Published:** 2025-08-01

**Authors:** JaeHui Song, HyunJi Lee, Youngshik Choe, Hyeyoung Lee

**Affiliations:** ^1^ Department of Applied Chemistry, Food Science and Technology Dong‐eui University Busan Republic of Korea; ^2^ Developmental Disorders & Rare Diseases Research Group Korea Brain Research Institute Daegu Republic of Korea; ^3^ Department of Food Science and Technology Dong‐eui University Busan Republic of Korea

**Keywords:** food, glycomics, mass spectrometry, nutrition

## Abstract

This review highlights advancements in mass spectrometry (MS)‐based glycomics in food and nutritional science. Carbohydrates, which are vital for human health, exhibit complex structures, making their analysis challenging. MS has become an indispensable tool for elucidating the structures of carbohydrates, including glycans, through soft ionization techniques such as MALDI and ESI. Furthermore, coupling MS with advanced separation techniques enhances sensitivity and resolution. This review underscores the pivotal contributions of Professor Carlito B. Lebrilla to glycomics research, particularly regarding milk oligosaccharides and dietary fibers, and their roles in gut health. Comprehensive food glycomic databases and MS‐based studies offer valuable insights into the functions of carbohydrates and their implications for nutrition.

**Abbreviations**
AIMS: ambient ionization mass spectrometry
BMO: bovine milk oligosaccharide
CASCADE: combined alcohol soluble carbohydrate determination
CAZyme: carbohydrate‐active enzyme
CE: capillary electrophoresis
CID: collision‐induced dissociation
DART: direct analysis in real‐time
DFG: Davis Food Glycopedia
DP: degree of polymerization
ECD: electron capture dissociation
EI: electron ionization
ESI: electrospray ionization
ETD: electron transfer dissociation
FDA: Food and Drug Administration
FITDOG: Fenton's initiation towards defined oligosaccharide groups
FTICR: fourier transform ion cyclotron resonance
GalNAc: N‐acetylgalactosamine
GC: gas chromatography
GI: gastrointestinal
GlcNAc: N‐acetylglucosamine
GSL: glycosphingolipid
HCD: higher‐energy collisional dissociation
HG: homogalacturonan
HILIC: hydrophilic interaction liquid chromatography
HMO: human milk oligosaccharide
HPAEC‐PAD: high‐performance anion exchange chromatography with pulsed amperometric detection
HPLC: high‐performance liquid chromatography
IMMS: ion mobility mass spectrometry
IMS: ion mobility spectrometry
IRMPD: infrared multiphoton dissociation
IT: ion trap
LACS: linkage analysis of carbohydrates
LTQ: linear ion trap
MACS: monomeric analysis of carbohydrates
MALDI: matrix‐assisted laser desorption/ionization
MS: mass spectrometry
MS/MS: tandem mass spectrometry
Neu5Ac: N‐acetylneuraminic acid
Neu5Gc: N‐glycolylneuraminic acid
NMR: nuclear magnetic resonance
NP: normal phase
PGC: porous graphite carbon
PMP: 1‐phenyl‐3‐methyl‐5‐pyrazolone
PNGase: peptide‐N‐glycosidase
Q: quadrupole
QIT: quadrupole ion trap
QqQ: triple quadrupole
RG: rhamnogalacturonan
RP: reversed‐phase
SCFA: short chain fatty acid
SPE: solid phase extraction
SRM: selected reaction monitoring
TMAH: tetramethylammonium hydroxide
TMS: trimethylsilyl
TOF: time‐of‐flight
UHPLC: ultrahigh performance liquid chromatography
USDA: United States Department of Agriculture
UVPD: ultraviolet photodissociation
XGA: xylogalacturonan

## 1 Introduction

Carbohydrates are the most abundant naturally occurring organic compounds on Earth and major food components. Carbohydrates exhibit a wide range of chemical and physical properties and various physiological effects in the human body (Damodaran 2017). Some carbohydrates form covalent bonds with other biomolecules, in which the carbohydrate components of glycoproteins and glycolipids are referred to as glycans (Varki et al. 2022). Recent advancements in mass spectrometry (MS) have led to in‐depth insights into carbohydrates from foods, including monosaccharides, oligosaccharides, polysaccharides, and glycans. Additionally, MS has made investigating the glycome (the complete collection of carbohydrates and glycans) as a whole (glycomics) possible. This review highlights the fundamental principles of MS in glycomics and gives detailed structures of carbohydrates discovered in various food matrices while providing insights into the impacts of carbohydrates on human health, which have been made possible through advancements in MS. In this context, the scientific achievements of Professor Carlito B. Lebrilla in this field are described. Professor Lebrilla has made significant contributions to advancing glycomics research through the development and application of cutting‐edge MS techniques. His pioneering work has provided comprehensive information regarding structural elucidation and functional analysis of carbohydrates and glycans, particularly in the context of food and human health.

### 1.1 Carbohydrates and Their Structural Characteristics

Carbohydrates are structurally characterized as polyhydroxylated aldehydes or ketones with the general formula C_x_(H_2_O)_y_, reflecting their designation as hydrates of carbon (Robyt 1998). Carbohydrates have highly diverse structural features, such as varying sizes, shapes, polarities and charges. They are usually classified according to their degree of polymerization (DP), as follows: monosaccharide (DP 1), disaccharide (DP 2), oligosaccharide (DP 3‐10), and polysaccharide (DP > 10) (Cummings and Stephen 2007).

Monosaccharides, the simplest carbohydrates, exhibit considerable structural diversity due to variations in factors such as the number of carbons, chirality, type of carbonyl group, ring structure, and chemical modifications. Naturally, monosaccharides typically contain five or six carbon atoms. The presence of chiral carbon atoms leads to multiple stereoisomers, meaning that monosaccharides with the same chemical formula can differ in the arrangement of their hydroxyl groups. For example, glucose, galactose, and mannose all have the formula C_6_H_12_O_6_ but vary in the orientation of their hydroxyl groups. Monosaccharides are further classified as aldoses or ketoses on the basis of the type of carbonyl group present. Monosaccharides can also form six‐membered pyranose or five‐membered furanose rings, and upon cyclization, an anomeric carbon is generated in one of two possible configurations, α and β. Additionally, chemical modifications significantly enhance the structural diversity of monosaccharides. Oxidation yields aldonic and uronic acids, while deoxygenation produces deoxy sugars such as fucose. The incorporation of amino groups forms amino sugars like glucosamine and galactosamine, which are often N‐acetylated to produce N‐acetylglucosamine (GlcNAc) and N‐acetylgalactosamine (GalNAc). Sialic acids, a family of nine‐carbon acidic sugars, add further complexity through their carboxyl and acetyl groups, with the most common forms being N‐acetylneuraminic acid (Neu5Ac) and N‐glycolylneuraminic acid (Neu5Gc).

Two monosaccharide units can be joined together by a glycosidic bond, which is the fundamental linkage among the monosaccharide building blocks of larger carbohydrates. A glycosidic bond is typically formed between the anomeric carbon of one monosaccharide and a hydroxyl group of another. As there are several hydroxyl groups in each monosaccharide, glycosidic linkages can vary, such as 1→2, 1→3, 1→4, and 1→6. Additionally, a monosaccharide can be involved in more than two glycosidic linkages, thus serving as a branchpoint. Carbohydrates have the distinct characteristic of branched sequences, which distinguish them from peptides and oligonucleotides, which typically have linear sequences. This unique feature plays a significant role in the structural diversity of carbohydrates. Glycosidic bonds can exist in either the α‐ or β‐linkage form, further increasing carbohydrate diversity. Larger carbohydrates consist of multiple monosaccharides linked together, ranging in size up to thousands of units, highlighting the extensive structural variety of these molecules.

A glycan is a carbohydrate structure composed of one or more monosaccharides linked together by glycosidic bonds. Glycans can exist as free entities, such as oligosaccharides and polysaccharides, or as parts of larger macromolecules. They are found in all living organisms, from bacteria to humans, and play essential roles in various biological processes (Stanley et al. 2022). Glycans are also widely present in foods derived from plants, animals, and microbes. When glycans are covalently attached to noncarbohydrate components, such as proteins or lipids, the resulting molecules are referred to as glycoconjugates. The main categories of glycoconjugates include glycoproteins (N‐glycans and O‐glycans), glycosphingolipids (GSLs), and proteoglycans. N‐Glycans are sugar chains attached to an asparagine (Asn) residue in a polypeptide chain and are found across all life forms, where they assist with protein folding, stability, and function. On the other hand, O‐glycans are sugar chains attached to the oxygen atom of a serine (Ser) or threonine (Thr) residue, often via N‐acetylgalactosamine (GalNAc). O‐Glycans are critical for processes such as cell‒cell communication, adhesion, signal transduction, immune surveillance, epithelial protection, and host‒pathogen interactions. GSLs, a type of glycolipid with a glycan attached to the lipid ceramide, are the primary glycolipids in animals and are commonly found in cell membranes. GSLs play key roles in cell signaling and membrane structure maintenance (Lowe and Marth 2003).

### 1.2 Diversity Among Food Carbohydrates

Carbohydrates play fundamental roles in both human nutrition and food science. As one of the primary macronutrients, carbohydrates serve as a major energy source for the human body, providing 4 kcal per gram. Glucose, the most basic monosaccharide, does not need to be digested and is fully absorbed in the intestine. Once it is delivered to the bloodstream, glucose enters the cytosol and undergoes glycolysis to generate energy (Campos et al. 2022). Most short carbohydrates contribute to sweetness and browning reactions, playing a positive role in enhancing the flavor and preference of diets (Fernstrom et al. 2012). Macromolecular carbohydrates exhibit wide structural diversity, and their digestion, absorption, and utilization by humans vary depending on their specific structures. For example, starch is hydrolyzed by human digestive enzymes into glucose, which serves as a vital energy source. In addition, starch is extensively utilized in food applications as a thickener, stabilizer, and gelling agent, highlighting its multifunctional role beyond nutrition. Dietary fibers are not digested by human digestive enzymes, but they do provide various health benefits, including improving gut health, reducing cholesterol levels, and controlling blood sugar (Elia and Cummings 2007; Scheppach et al. 2001).

**Figure 1:**
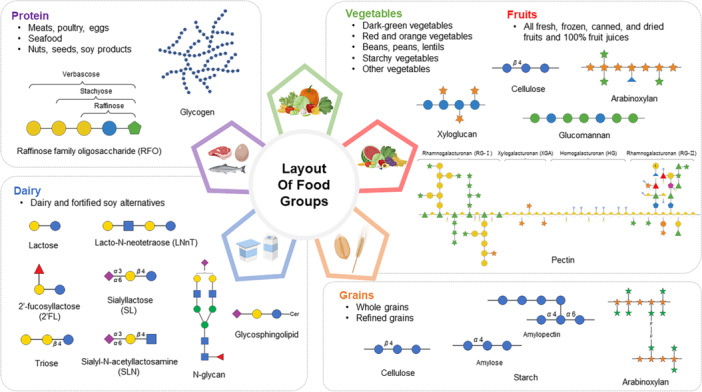
Carbohydrate structures found in various food groups classified according to the USDA MyPlate guidelines. [Color figure can be viewed at wileyonlinelibrary.com]

According to the USDA MyPlate guidelines, different types and amounts of carbohydrates are found in the various food groups (Figure 1). For example, a variety of complex polysaccharides make up the cell walls of vegetables and fruits. These include cellulose, hemicellulose, lignin, and pectin (Padayachee et al. 2017). Cellulose is a linear polymer of glucose units linked by β‐glycosidic bonds, whereas hemicellulose consists of diverse monosaccharides, such as xylose, mannose, glucose, galactose, arabinose, galacturonic acid, and glucuronic acid. The composition of hemicellulose varies depending on its polymeric structure (Rao et al. 2023). Pectin, another key polysaccharide, is rich in covalently bonded galacturonic acid residues, with homogalacturonan (HG) being its main component. Pectin also contains polysaccharides with specialized structures, such as xylogalacturonan (XGA), rhamnogalacturonan I (RG‐I), and rhamnogalacturonan II (RG‐II) (Mohnen 2008).

Grains, which include barley, brown rice, oats, white bread, and cereal grains, serve as staple foods and are an important source of carbohydrates. Starch, the primary carbohydrate in grains, consists of amylose and amylopectin, both of which are glucose polymers. Starch comprises 90% of the dry weight of milled rice, functioning as a carbohydrate reservoir and an excellent source of dietary energy (Patindol et al. 2015). Wheat grains are composed of approximately 65%–75% carbohydrates, of which 65%–75% are typically starch. Additionally, wheat grains contain approximately 10% cell wall polysaccharides, such as cellulose, arabinoxylan, and β‐glucan (Lafiandra et al. 2014).

Dairy products, such as bovine milk, are important sources of carbohydrates, among which lactose is the primary carbohydrate. Lactose, a disaccharide composed of glucose and galactose, serves as an essential energy source, particularly for infants and young mammals. In addition to lactose, bovine milk contains a variety of oligosaccharides that are structurally similar to human milk oligosaccharides (HMOs), although their abundance and composition differ. Bovine milk oligosaccharides (BMOs) and HMOs are derived from lactose and can include monosaccharides such as glucose, galactose, fucose, and Neu5Ac arranged in a variety of diverse configurations. Among them, 2ʹ‐fucosyllactose (2ʹ‐FL) is the most abundant oligosaccharide in human milk, whereas 3’‐sialyllactose (3ʹ‐SL) is predominate in bovine milk (Zivkovic and Barile 2011). N‐Glycans and GSLs are also present in milk.

Carbohydrates are present in much lower quantities in protein‐rich foods, such as meat and fish, than in other types of foods. Carbohydrates are primarily stored in animal foods as glycogen, which is found in muscle tissues. Compared with mammalian muscle, fish muscle contains lower glycogen levels, with red muscle having a higher glycogen content than white muscle does (Zhang et al. 2017). Legumes, such as soybeans, are known for their abundance of galactooligosaccharides, including raffinose, stachyose, and verbascose, which consist of galactose units linked to a terminal sucrose unit. In addition, a variety of polysaccharides, such as cellulose, pectin, and hemicelluloses, along with galactans, xyloglucans, and arabinogalactan, are abundant in soybeans (Karr‐Lilienthal et al. 2005).

This diverse distribution of carbohydrates across food groups highlights their multifaceted roles in nutrition, ranging from energy provision to having certain functions in food products. Understanding these variations is essential for designing dietary recommendations and developing food formulations that cater to individuals with specific nutritional needs.

### 1.3 Classical Carbohydrate Analysis Methods

Carbohydrates, despite their essential role in human health and nutrition, have historically been challenging to analyze owing to the complexity and diversity of their chemical structures. The intricacies in carbohydrate composition have led to difficulties in determining their precise quantities, types, and classifications. In accordance with the US Food and Drug Administration (FDA) nutrition labeling regulations, the carbohydrate content is detailed as total carbohydrates, dietary fiber, total sugars, and added sugars (Metzger and Nielsen 2017). The total carbohydrate content is determined indirectly by subtracting the weights of protein, fat, moisture, and ash from the total food weight rather than through direct measurement. Dietary fiber is typically measured gravimetrically after the digestible components are removed, but variations in fiber structure further complicate accurate fiber quantification (DeVries 2004).

Early methods, such as colorimetric tests, relied on chemical reactions between carbohydrates and specific reagents to produce a color change, which could then be quantified. For example, the phenol‒sulfuric acid method remains one of the simplest and most reliable techniques for quantifying total sugars. Another method, the Fehling test, detects reducing sugars on the basis of the reduction of copper(II) to copper(I), indicating the presence of free aldehyde or ketone groups in sugars. While useful, these early techniques have limitations, particularly in separating complex mixtures of carbohydrates or providing detailed structural information (Kurzyna‐Szklarek et al. 2022).

With advancements in separation technology, more sophisticated instruments have revolutionized carbohydrate analysis. Techniques such as gas chromatography (GC) and high‐performance liquid chromatography (HPLC) have significantly improved the separation and quantification of sugars in complex samples. High‐performance anion exchange chromatography with pulsed amperometric detection (HPAEC‐PAD) has further increased the sensitivity and resolution of carbohydrate analysis, enabling the detection of low‐abundance carbohydrates. Nuclear magnetic resonance (NMR) spectroscopy has also been instrumental in carbohydrate analysis, allowing for the elucidation of complex carbohydrate structures, including the elucidation of branching points and stereochemistry (Herrero et al. 2011). Despite these advancements, carbohydrate analysis continues to pose challenges due to the wide diversity of carbohydrate structures in nature.

### 1.4 In‐Depth Analysis of Food Carbohydrates and Glycans Using MS

Carbohydrates and glycans play diverse roles in biological systems, extending beyond their function as an energy source. Non‐starch polysaccharides (dietary fiber) are not digested by human digestive enzymes; instead, they traverse the gastrointestinal (GI) tract and reach the large intestine, where the majority of the gut microbiota resides. Gut microbes possess diverse carbohydrate‐active enzymes (CAZymes) that breakdown dietary fiber into short‐chain fatty acids (SCFAs), which contribute to a healthy immune response (Wardman et al. 2022). Specific carbohydrates can selectively promote the growth of beneficial gut bacteria while suppressing harmful strains, contributing to overall gut health. In addition, glycans act as recognition molecules in various biological processes, including host‒microbe interactions, and play roles in microbial attachment, invasion, and symbiosis (Payling et al. 2020).

Characterizing the complex structures of food carbohydrates and glycans is essential for understanding their specific functions. However, this presents analytical challenges. Advancements in techniques such as MS have significantly improved our ability to characterize and analyze carbohydrates and glycans, leading to a better understanding of their diversity and biological implications. First, MS provides detailed structural information about carbohydrates, including their molecular weights, monosaccharide compositions, sequences, linkage patterns, anomeric configurations, branching patterns, and modifications. This level of detail is crucial for understanding how carbohydrate structures are related to their specific functions, such as interactions with enzymes or receptors. Second, MS is highly sensitive and capable of detecting and quantifying carbohydrates present in trace levels. Such sensitivity is particularly important when analyzing complex samples such as biological samples, where target carbohydrates may be present in low abundance. Additionally, modern MS techniques, especially when coupled with separation methods such as liquid chromatography (LC) or high‐throughput sample preparation, enable the rapid analysis of many samples. This high‐throughput capability is essential for studies involving large sample sets, clinical trials, or the analysis of complex food products containing diverse carbohydrate profiles (Ashline et al. 2007; Zaia 2010).

## 2 Advances in MS‐Based Methods for Food Carbohydrate Analysis

Owing to its ability to provide detailed molecular and structural information, MS has emerged as a pivotal tool in carbohydrate analysis in food. Advances in MS‐based methods, including the development of soft ionization techniques and high‐resolution mass analyzers, have greatly increased our ability to characterize carbohydrates precisely. In this article, the latest innovations in MS instrumentation, ionization methods, and separation techniques are explored, with a focus on their applications in the analysis of food carbohydrates at the monosaccharide, oligosaccharide, polysaccharide, and glycan levels.

### 2.1 Carbohydrate Analysis by MS: Key Instrumentation and Techniques

A mass spectrometer generally consists of three main components: an ionizer, an analyzer, and a detector. The ionizer converts the sample into ions, and the analyzer separates these ions on the basis of their mass‒charge ratio (*m/z*). With the development of soft ionization methods such as matrix‐assisted laser desorption/ionization (MALDI) and electrospray ionization (ESI) in the late 1980s, MS became a widely adopted technique for analyzing biomolecules (Harvey 2017). Unlike earlier ionization methods such as electron ionization (EI), soft ionization techniques allow for the ionization of molecules without significant fragmentation, preserving the structural integrity of delicate molecules such as carbohydrates.

ESI, in particular, is a soft ionization method where a solution of a sample is passed through a charged capillary needle under high voltage, producing a spray of charged droplets. As the solvent evaporates, these droplets shrink, ultimately forming gas phase ions. ESI is favored for carbohydrate analysis because it generates intact molecular ions with minimal fragmentation, making it ideal for analyzing large and complex carbohydrates. However, ESI can produce multiple charged ion adducts, complicating data analysis, and its performance is sensitive to the pH of the analyte and the presence of salts, which can affect ionization efficiency (Fenn et al. 1989; Grabarics et al. 2021). MALDI, another soft ionization method commonly used in carbohydrate analysis, is a method in which the sample is mixed with a matrix—an organic molecule that absorbs laser energy. Upon laser irradiation, the matrix transfers energy to the sample, causing the ionization and desorption of the analyte. One advantage of MALDI is its ability to analyze high‐molecular‐weight carbohydrates, but the in‐source fragmentation of unstable groups, such as sialic acids, can also occur (Harvey 2025; Wang et al. 2023).

Once analytes are ionized, they enter the analyzer and are separated by *m/z*. The commonly used analyzers for carbohydrate analysis are quadrupole (Q), time‐of‐flight (TOF), ion trap (IT), orbitrap, and Fourier transform ion cyclotron resonance (FTICR). These analyzers can be broadly classified as single or hybrid analyzers. Quadrupole mass analyzers use four parallel rods into which a combination of DC and RF voltages is applied. The electric field created by the rods allows only ions of a specific *m/z* to pass through the analyzer and reach the detector at a given time, filtering out other ions. Quadrupole analyzers are known for their simplicity, robustness, and relatively low cost, but they have limitations in m/z range, mass accuracy, and resolution (Glish and Vachet 2003). TOF analyzers determine the m/z of ions by measuring the time required for them to travel through a field‐free flight tube after being accelerated by an electric field. During acceleration, ions with the same charge state are imparted with the same kinetic energy. As a result, lighter ions travel faster and reach the detector before heavier ones. TOF analyzers offer high sensitivity, a wide m/z range, and rapid spectral acquisition, making them well‐suited for profiling complex glycan mixtures (Guilhaus et al. 1997). Ion trap analyzers trap ions in a three‐dimensional electric field before selectively releasing them into the detector on the basis of their *m/z*. Orbitrap analyzers trap ions in an electrostatic field between a central spindle electrode and a barrel‐like outer electrode, where the ions oscillate around the spindle while also moving back and forth along its axis. The frequency of these oscillations is used to determine the *m/z* ratio. Orbitrap analyzers provide high sensitivity and high mass accuracy (Hardman and Makarov 2003; Senyuva et al. 2015). FTICR mass analyzers trap ions in a strong magnetic field, causing them to move in circular paths, and the frequency of these circular motions is inversely proportional to their *m/z* ratio. FTICR offers the highest mass accuracy and resolution among all mass analyzers (Nikolaev et al. 2016).

Hybrid mass analyzers combine two or more mass analyzers to provide more information. These analyzers first isolate ions of a specific *m/z* (precursor ions) using one mass analyzer, after which these ions are fragmented and subsequently analyzed by the second mass analyzer. Q‐TOF instruments combine a quadrupole mass analyzer with a TOF analyzer and offer high sensitivity and mass accuracy and the ability to perform tandem mass spectrometry (MS/MS) experiments, making Q‐TOF instruments suitable for analyzing complex carbohydrate mixtures and determining the carbohydrate structures (Sun et al. 2015; Wu et al. 2017). Triple quadrupole (QqQ) analyzers employ three quadrupoles in series. The first and third quadrupoles act as mass filters, whereas the second quadrupole functions as a collision cell. This setup enables the performance of selected reaction monitoring (SRM) experiments, which are highly specific and sensitive for quantifying known carbohydrates (Fong et al. 2011; Xu et al. 2018). Quadrupole ion trap (QIT) instruments use quadrupolar electric fields to trap ions in space, enabling their sequential isolation and fragmentation. These instruments can perform multiple stages of fragmentation (MS^n^). Linear ion trap (LTQ)‐Orbitrap instruments combine a linear ion trap with an orbitrap analyzer, offering high sensitivity, mass accuracy, and resolution and MS^n^ capabilities for complex carbohydrate analysis (Rohmer et al. 2011; Senyuva et al. 2015).

MS/MS, which involves multiple stages of mass analysis, is essential for determining the structure of carbohydrates. MS/MS methods fragment selected ions to provide detailed information about their structure. Collision‐induced dissociation (CID) is a widely used MS/MS technique in which selected precursor ions are accelerated into a collision cell filled with an inert gas (e.g., argon). The collisions with the gas molecules transfer energy to the ions, causing them to fragment. CID is particularly useful for determining the sequence of monosaccharide units and identifying the branching patterns of carbohydrates. CID typically produces B/Y‐ and C/Z‐type fragment ions, which arise from the cleavage of glycosidic bonds connecting adjacent sugar residues (Domon and Costello 1988; Tang et al. 2018). Higher‐energy collisional dissociation (HCD) is another collision‐induced dissociation technique similar to CID that operates at higher energies. This increased energy leads to more extensive fragmentation, complementing the structural information provided by CID, particularly for differentiating isomeric carbohydrates from cross‐ring cleavage within the sugar ring of a monosaccharide (Gray et al. 2019; Kailemia et al. 2014; Wang et al. 2021). Electron capture dissociation (ECD) and electron transfer dissociation (ETD) are particularly valuable for analyzing sulfated carbohydrates, which are often challenging to characterize using conventional fragmentation methods. ECD and ETD involve the transfer of an electron to the precursor ion, leading to fragmentation along the carbohydrate backbone. These methods provide complementary information to CID and HCD and are particularly useful for determining the positions of sulfate groups in carbohydrates (Han and Costello 2011). Infrared multiphoton dissociation (IRMPD) uses infrared photons to fragment ions. IRMPD can differentiate between isomeric carbohydrates, providing insights into linkage positions and anomeric configurations (Guerrero and Lebrilla 2013; Li et al. 2009).

Recent advances in mass spectrometry have introduced a diverse set of radical‐driven fragmentation techniques that provide improved structural information for complex glycans. These approaches address limitations of conventional CID and HCD, particularly for isomer resolution and labile modification retention. Ultraviolet photodissociation (UVPD) uses high‐energy photons to induce extensive fragmentation, including glycosidic and cross‐ring cleavages, making it highly effective for linkage analysis and localization of sulfate or acyl modifications. UVPD has been applied to glycopeptides, bacterial oligosaccharides, and human milk oligosaccharides (HMOs), often providing greater sequence coverage than CID (Brodbelt et al. 2019). Electron‐based dissociation techniques such as electron capture dissociation (ECD) and electron transfer dissociation (ETD) generate radical‐driven fragments along the carbohydrate backbone while preserving labile groups. These methods are particularly useful for analyzing sulfated glycans and glycopeptides (Yu et al. 2013; Grabarics et al. 2022). Electronic excitation dissociation (EED) and electron‐induced dissociation (EID) enable high‐energy fragmentation of singly charged ions, producing rich cross‐ring and glycosidic cleavages without charge reduction, and have been applied to tumor antigens, bacterial oligosaccharides, and isomeric milk glycans (Yu et al. 2013; Grabarics et al. 2022). Electron detachment dissociation (EDD) and negative electron transfer dissociation (NETD) are powerful tools for sulfated glycosaminoglycans, providing enhanced retention of sulfate groups and abundant diagnostic fragments. Charge transfer dissociation (CTD) uses helium cation collisions to produce fragmentation patterns useful for linkage determination in neutral and acidic oligosaccharides (Grabarics et al. 2022; Harvey 2023). Finally, chemical derivatization strategies such as free radical activated glycan sequencing (FRAGS) and radical‐directed dissociation (RDD) generate targeted fragmentation via radical precursors or photolabile tags. These methods have been applied to distinguish isomeric disaccharides, glycosphingolipids, and HMOs with high specificity (Brodbelt et al. 2019; Grabarics et al. 2021; Wang et al. 2021).

A mass analyzer for carbohydrate analysis should be selected on the basis of the specific application and the type of information needed. While accurate mass measurements obtained from single‐stage MS experiments provide insight into carbohydrate composition, their integration with various methodologies—such as fragmentation techniques and chemical derivatization—facilitates the elucidation of additional structural details. For applications demanding high‐throughput analysis, TOF instruments are commonly favored. Conversely, hybrid analyzers, such as Q‐TOF, QqQ, and LTQ‐Orbitrap, are better suited for the elucidation of detailed structural information.

While MS is powerful tool for identifying and quantifying carbohydrates, separation techniques often need to be applied before MS analysis. This combined approach offers several advantages. By separating complex mixtures before MS analysis, the suppression of the ions of less abundant components by more abundant components is reduced, leading to increased sensitivity and the ability to detect low‐abundance carbohydrates. In addition, separation techniques excel in terms of separating carbohydrate isomers on the basis of their physicochemical properties. Such separation simplifies the obtained MS spectra and allows for the identification and characterization of individual isomers from complex mixtures. HPLC, capillary electrophoresis (CE), GC, and ion mobility spectrometry (IMS) are used for separating carbohydrates and glycans. By utilizing separation techniques specifically designed for different carbohydrate classes, the sensitivity and resolution of the MS analyses can be increased, enabling more precise structural characterization of carbohydrates and glycans. The following section introduces MS analysis methods for classifying food carbohydrates according to their DP. A summary is provided in Table 1.

**Table 1:** Characteristics of mass spectrometry (MS)‐based analysis of carbohydrates by type.

Separation method	MS	Derivatization	Characteristic(s)	Reference(s)
Monosaccharides				
GC	EI–Q	Yes	Traditional method	Ačanski and Vujić (2014)
LC (HILIC)	ESI–QqQ	No	No need for derivatization; poor chromatographic resolution	Ghfar et al. (2015); Han et al. (2020)
LC (RP)	ESI–QqQ	Yes	Excellent sensitivity; high chromatographic resolution	Honda et al. (1989); Song et al. (2024); Xu et al. (2018)
Disaccharides				
LC (PGC)	ESI–IT	No	Excellent performance in separating various isomers	Martín‐Ortiz et al. (2020)
LC (HILIC)	ESI–IT	No	Separation based on polymerization and monomer units	Martín‐Ortiz et al. (2020)
None	ESI–IMMS	No	Isomers can be distinguished through their unique mobility profiles	Li et al. (2013)
None	DART–AIMS–Orbitrap	Yes	Rapid and simple differentiation of isomers in complex matrices	Ren et al. (2023)
Oligosaccharides				
None	MALDI–Orbitrap or FTICR	Yes or no	High mass accuracy with excellent fragmentation prospects	Barboza et al. (2009); Ninonuevo et al. (2007); Park and Lebrilla (2005); Rohmer et al. (2011)
LC (PGC)	ESI–Q‐TOF	No	Effective separation of isomers and large‐scale profiling using accurate mass and retention time	Lee, Jo et al. (2024); Song et al. (2022); Wu et al. (2012)
LC (PGC)	ESI–Orbitrap	Yes or no	Effective separation of isomers; high mass accuracy	Lin et al. (2022); Oursel et al. (2017)
LC (PGC)	ESI–QqQ	No	Effective separation of isomers; absolute quantification; enhanced specificity	Balogh et al. (2015); Hong et al. (2014)
LC (HILIC)	ESI–Orbitrap	No	High mass accuracy; excellent fragmentation	Liu et al. (2014); Remoroza et al. (2018)
LC (HILIC)	ESI–QqQ	No	Absolute quantification; enhanced specificity	Fong et al. (2011); Prieto‐Santiago et al. (2022); Zhang et al. (2019)
Polysaccharides				
Monosaccharide composition				
GC	EI–Q	Yes	Determination of various monosaccharide forms (anomers and ring structures)	Doco et al. (2001); Xia et al. (2018)
LC (RP)	ESI–QqQ	Yes	Excellent sensitivity; high reliability for quantification; short analysis time	Amicucci, Galermo, Guerrero et al. (2019); Amicucci, Galermo, Nandita et al. (2019); Huang et al. (2025); Pasha and Ahmad (2021)
LC (HILIC)	ESI–QqQ	No	No need for derivatization; poor chromatographic resolution	Gao et al. (2011)
Sequencing				
LC (PGC)	ESI–Q‐TOF	No	Precise sequence identification	Amicucci, Galermo, Guerrero et al. (2019); Castillo et al. (2021)
LC (HILIC)	ESI–Orbitrap	No	Precise sequence identification	Liu et al. (2018)
Glycosidic linkage				
LC (RP)	ESI–QqQ	Yes	Detection of a wide variety of glycosidic linkage patterns; relative quantification; derivatization required	Amicucci, Galermo, Guerrero et al. (2019); Galermo et al. (2018, 2019)
LC (HILIC)	ESI–Q‐IT	No	Analysis of complex glycosidic linkages without the need for derivatization	Juvonen et al. (2019); Ognyanov et al. (2020)
Fingerprinting				
None	MALDI–TOF	No	Minimal sample preparation; effective for high molecular weight compounds; no need for derivatization; poor quantification accuracy	Pandeirada et al. (2022); Qu et al. (2019); Westphal et al. (2010)
LC (PGC)	ESI–Q‐TOF	Yes	Accurate quantitative and qualitative analysis of polysaccharides with high‐throughput FITDOG method; more comprehensive characterization of polysaccharide types	Amicucci et al. (2020); Bacalzo et al. (2022); Nandita et al. (2021)

### 2.2 MS Analysis of Monosaccharides and Disaccharides

Monosaccharides, the simplest sugars and fundamental units of carbohydrates, present a considerable analytical challenge in MS detection because of the presence of isomers with identical molecular weights but different hydroxyl group arrangements. While MS can differentiate monosaccharides on the basis of their unique *m/z*—such as hexose (*m/z* 162.2), pentose (*m/z* 132.0), N‐acetylhexosamine (*m/z* 203.1), deoxyhexose (*m/z* 146.1), and N‐acetylneuraminic acid (*m/z* 291.1)—it cannot resolve isomers sharing the same chemical formula. For example, eight D‐aldohexoses and eight D‐ketohexoses remain indistinguishable using MS alone. To overcome this limitation, MS is often coupled with separation techniques such as LC, GC, IMS, or CE. These methods enable the resolution of epimers and other structural isomers before MS detection, significantly enhancing the accuracy and reliability of monosaccharide analysis.

GC‒MS has been widely used for analyzing monosaccharides since the 1960s (Björndal et al. 1967; Sawardeker et al. 1965). Because monosaccharides are not sufficiently volatile for GC analysis, they must be derivatized before being injected into the GC‒MS system. Common methods include formation of alditol acetates, which is well established but time‐consuming and loses anomeric configuration. Aldononitrile acetates offer faster derivatization and single peaks per sugar, but are unsuitable for ketoses and less reliable for quantification. Trimethylsilyl (TMS) ethers improve volatility but produce multiple peaks due to tautomerization. TMS oximes (TMSO) simplify chromatograms by limiting peak multiplicity and are stable and broadly applicable (Ruiz‐Matute et al. 2011). Monosaccharides are identified and quantified by analyzing the specific fragment ions in the mass spectra generated by EI, along with the corresponding GC retention times. Despite its usefulness, this technique has limitations, including poor sensitivity and dynamic range, labor‐intensive sample preparation, and prolonged analytical times, making it less suitable for high‐throughput analysis.

Recent advancements in combining HPLC or ultrahigh‐performance liquid chromatography (UHPLC) with MS have overcome these limitations. In terms of LC separation, hydrophilic interaction liquid chromatography (HILIC) and reversed‐phase (RP) C18 chromatography are the most widely used methods. As native monosaccharides are polar, HILIC is the suitable stationary phase for their separation (Alpert 1990). HILIC offers the advantage of analyzing monosaccharides directly without the need for derivatization. However, it also has limitations regarding the simultaneous analysis of a diverse range of monosaccharides and achieving complete separation (Han et al. 2020; Liu et al. 2021). The C18 stationary phase better separates analytes on the basis of their hydrophobicity; therefore, when analyzing monosaccharides, derivatization is employed to increase hydrophobicity. Among the various derivatization methods, the most commonly used is 1‐phenyl‐3‐methyl‐5‐pyrazolone (PMP) derivatization, developed by Honda et al. (1989), wherein the active methylene group of PMP reacts with the reducing end of a monosaccharide, facilitating the attachment of the pyrazolone ring to the monosaccharide molecule. PMP labeling can be employed to analyze carbohydrates containing acid‐labile groups without causing desialylation or isomerization. Additionally, compared with their nonderivatized counterparts, PMP derivatives generate more intense MS signals (Honda et al. 2003). HPLC‐ESI‐MS is frequently used with QqQ in SRM mode, which enhances selectivity and sensitivity for target monosaccharide analysis (Xu et al. 2018). However, this technique has certain drawbacks. For example, the reaction conditions for the derivatization procedure may require careful optimization to ensure complete derivatization. Additionally, this process might not be suitable for all monosaccharide types, especially those with specific structural features that do not readily react with PMP, such as ketose and sugar alcohol moieties (Wang et al. 2018).

Disaccharides, including maltose, lactose, and sucrose, are important ingredients in the food and agricultural industries. Disaccharide complexity arises from the structural diversity of their constituent monosaccharides and the specific ways in which these monosaccharides are linked through glycosidic bonds. Such diversity is driven by the variety of monosaccharides (e.g., glucose, fructose, galactose, and mannose), as well as the anomeric configurations (α or β) of these sugars. Additionally, the position and type of glycosidic linkage, such as α(1→4) in maltose, β(1→4) in lactose, or α(1→2) in sucrose, significantly influence the chemical and functional properties of disaccharides. Native disaccharides can be analyzed via LC‒MS. Porous graphitic carbon (PGC) is useful for separating isomers, whereas HILIC separates isomers on the basis of their polymer and monomeric units (Ehlers Cheang et al. 2024; Martín‐Ortiz et al. 2020). Furthermore, MS/MS is crucial for determining glycosidic linkages and disaccharides sequences via analysis of the fragment ions, and SRM mode is used to increase sensitivity. Without LC separation, ion mobility mass spectrometry (IMMS) resolves disaccharide isomers with different structural configurations by measuring their cross‐sectional areas (Li et al. 2013). Recently, ambient ionization tandem mass spectrometry (AIMS) with in situ methylation, which uses a direct analysis in real‐time (DART) ion source, has enabled the rapid methylation and ionization of disaccharides with tetramethylammonium hydroxide (TMAH). This technique generates methylated ions that are analyzed by MS and MS/MS, producing distinct fragmentation patterns for identifying structural isomers. The method is fast, robust, and requires minimal sample preparation, making it suitable for analyzing disaccharide isomers in commercial products (Ren et al. 2023).

### 2.3 MS Analysis of Oligosaccharides

Techniques in MS for oligosaccharide analysis are rapidly evolving and have shown significant improvements. EI can be used to ionize smaller oligosaccharides; however, MALDI and ESI are the more frequently employed ionization methods, as they enable the ionization of intact molecules (Kailemia et al. 2014). During the MALDI process, the sample is first dissolved in an organic solvent and then mixed with a matrix solution, such as dihydroxybenzoic acid. The resulting mixture is then dried and applied as a thin layer on a target plate. Upon exposure to an intense laser beam, the dried sample absorbs energy from the laser, leading to ionization. This process typically yields mass spectra with predominantly singly charged ions, such as [M+Na]^+^ and [M+H]^+^ ions in positive mode or [M‐H]^−^ ions in negative mode. MALDI is well suited for analyzing samples with high concentrations of salt, which is a great advantage when studying various biological samples. MALDI also has excellent ionization efficiency, even at relatively high mass ranges, making it a valuable tool for oligosaccharide analysis. ESI operates by directing a solution through an ion source, in which the sample is ionized. This process can yield mass spectra with multiple charged ions, especially when analyzing large oligosaccharides, which allows the use of mass spectrometers with limited *m/z* detection ranges. Nano‐ESI offers significant advantages over conventional ESI for oligosaccharide analysis, including increased sensitivity and reduced sample consumption (Juraschek et al. 1999; Karas et al. 2000). In addition, permethylation of oligosaccharides enhances the detection sensitivity of both neutral and acidic oligosaccharides (Ruhaak et al. 2010; Wuhrer 2013).

Once oligosaccharides are ionized, they are analyzed by mass analyzers. TOF analyzers separate ions on the basis of their time of flight after acceleration in an electric field, whereas Q‐TOF instruments are hybrid quadrupole and TOF analyzers that provide MS/MS capabilities. Q‐TOF analyzers have an increased mass range, making them suitable for analyzing both small and large oligosaccharides. The acquisition rate can reach several hundred spectra per second, and upon combination with HPLC, better quantitative results are possible (Wu et al. 2017). Ion traps are another type of mass analyzer. The geometry of quadrupole traps enables molecular weight determination, with fragmentation occurring directly within the analyzer. Ion selection for fragmentation is achieved by ejecting all ions except the target ion. The Orbitrap is one of the most advanced high‐resolution mass analyzers, offering exceptional resolving power—typically up to 500,000 at m/z 200. In hybrid Orbitrap instruments, this high resolution is combined with MSⁿ capabilities provided by integrated ion traps, making them particularly well suited for precise oligosaccharide analysis, including the characterization of structural isomers (Lin et al. 2022; Liu et al. 2014; Rohmer et al. 2011). FTICR instruments are also high‐resolution mass analyzers that provide the greatest measurement accuracy and resolving power currently achievable. In addition to their analytical precision, FTICR platforms are uniquely suited for advanced electron‐based dissociation techniques such as ECD, enabling rich fragmentation patterns and detailed structural characterization of complex glycans (Park and Lebrilla 2005). Ion mobility spectrometry (IMS) adds a valuable dimension to oligosaccharide analysis by separating isomeric species based on their size, shape, and charge. It is especially useful for distinguishing linkage and positional isomers, such as α2,3‐versus α2,6‐sialylation and different fucosylation patterns. When coupled with LC–MS and MS/MS workflows, IMS enhances isomer resolution and supports detailed structural characterization, even in the absence of authentic standards (Manz et al. 2022; Bansal et al. 2023).

Using the accurate masses obtained from MS, the DPs and monosaccharide compositions can be easily determined. MS/MS involves fragmenting oligosaccharide ions and analyzing the resulting fragments to determine their sequence and branching (Zhao et al. 2011). Different types of fragment ions provide information about oligosaccharide sequences and linkages, and MSⁿ techniques have proven especially useful for identifying branching sites and linkage positions in complex oligosaccharides (Ashline et al. 2005; Ashline et al. 2007; Liew et al. 2021; Kouzounis et al. 2022). Glycomic databases such as Glycomics@ExPaSy (https://www.expasy.org/search/glycomics) have aided in analyzing and understanding oligosaccharide structures (Liu et al. 2023). Additionally, enzymatic methods use exoglycosidases to selectively cleave terminal monosaccharide residues, which, when used in series, can reveal the sequence and linkages of an oligosaccharide (Aldredge et al. 2013; Wu et al. 2010).

Oligosaccharides can be analyzed with or without the integration of separation techniques. While MALDI is not typically coupled online with separation methods, EI is almost always paired with GC, and ESI is commonly linked to liquid‐phase separation techniques such as HPLC and CE. The structural complexity of oligosaccharides results in a wide range of isomers, making separation techniques essential to accurately characterize their diverse forms and reduce ion suppression. EI is consistently coupled with GC, as EI provides fragmentation data for identifying glycosidic linkages, whereas GC retention times aid in the simultaneous determination of monosaccharide composition. However, carbohydrates require derivatization to become volatile, which is needed before GC‒MS injection. However, this approach has notable limitations. Glycosidic linkages involving uronic acids are challenging to hydrolyze efficiently, making uronic acids undetectable as alditol acetates. Additionally, the reduction step leads to the loss of the anomeric configuration of glycosyl residues. Another drawback is that the GC‒MS analysis process is typically lengthy due to the extended GC separation time and labor‐intensive sample preparation (Dong and Fang 1995; Hernández‐Hernández et al. 2011).

In the case of ESI, several LC stationary phases are widely employed, such as RP, HILIC, and PGC. RP chromatography, such as with C18 columns, is used for derivatized (permethylation or perbenzoylation) oligosaccharides, which become nonpolar (Chaturvedi et al. 1997). HILIC is particularly well suited for analyzing highly polar compounds such as derivatized or nonderivatized oligosaccharides. In this technique, the hydrophilic stationary phase retains polar analytes, while the mobile phase typically consists of a high content of organic solvent, allowing for better solubility of the oligosaccharides. HILIC provides greater selectivity in oligosaccharide analysis than RP chromatography does and is particularly useful for separating complex glycan isomers that vary in terms of position and linkage (Nagy et al. 2017). HILIC can also effectively separate isoforms of sialic acid, a component of many oligosaccharides (Yan et al. 2018). A common labeling reagent used in this technique is 2‐aminobenzamide (2‐AB), which aids in detection (Česla et al. 2016; Marino et al. 2011). PGC is a powerful method for oligosaccharide analysis, as it engages in both hydrophobic and surface interactions with oligosaccharides, and ionic interactions also play a role in their retention (Russo et al. 2024). PGC is particularly effective for distinguishing between various isomers, including linkage isomers and α/β anomeric forms. PGC stands out for its isomer resolution capabilities, making it a powerful tool for comprehensive oligosaccharide analysis (Balogh et al. 2015; Niñonuevo et al. 2005). When coupled with MS, PGC provides additional separation, offering unparalleled potential for the detailed characterization of oligosaccharides and glycans. Notably, PGC has been successfully applied to analyze native and reduced oligosaccharides in diverse samples, including milk and fermented foods (Song et al. 2022; Wu et al. 2017).

In addition to LC‐based separations, capillary electrophoresis (CE) has also been employed for oligosaccharide analysis, particularly when coupled with mass spectrometry (CE–MS). CE offers outstanding separation efficiency, making it especially valuable for resolving isomeric glycan structures that are difficult to distinguish by MS alone. CE–MS approaches—often using derivatization strategies—enable high‐sensitivity detection and isomer resolution for oligosaccharides (Mechref and Novotny 2009; Coenen et al. 2008).

### 2.4 MS Analysis of Polysaccharides

Polysaccharides are macromolecular carbohydrates in which the monosaccharide units are covalently linked by glycosidic bonds and typically consist of hundreds to thousands of monosaccharide units linked together. Polysaccharides are widely distributed in nature and are highly valuable in various industrial sectors because of their diverse applications. The complexity of polysaccharides arises from the diversity of monomeric units and linkage types, making their structural elucidation challenging, necessitating extensive research. Recent advancements in MS technologies have greatly improved the ability to analyze a range of polysaccharide structures, providing deeper insights into their intricate architectures.

Polysaccharides generally cannot be directly analyzed by MS because of their low ionization efficiency. Therefore, they are often depolymerized into smaller oligosaccharides and monosaccharides using enzymatic or chemical processes. For example, enzymatically released plant oligosaccharides have been utilized as diagnostic fingerprints to identify polysaccharides, with MALDI‐TOF MS widely employed for their characterization, as both MALDI and TOF MS have shown advantages in the analysis of high‐molecular‐weight compounds (Lerouxel et al. 2002; Wang et al. 2023). Despite its utility, this approach typically provides only partial structural information. In particular, when MALDI‐MS is used without prior separation techniques such as LC, different oligosaccharide or disaccharide isomers appear as a single peak, making it difficult to distinguish them—even when MS/MS is applied—due to the limited fragmentation specificity in the absence of chromatographic separation. Furthermore, enzymatic hydrolysis requires specific enzymes tailored to each polysaccharide type, as no universal enzyme exists that can effectively cleave all polysaccharides. The monosaccharide composition is determined by acid hydrolysis followed by GC‒MS or HPLC‒MS/MS analysis to infer the structure of the parent polysaccharide (Doco et al. 2001; Xia et al. 2018). However, this method disrupts the original arrangement of monosaccharide units, resulting in the loss of structural information and making it less suitable for comprehensive polysaccharide identification.

Recently, the Carlito group developed a method for elucidating polysaccharide structures known as the fingerprinting approach. The Fenton's initiation toward defined oligosaccharide groups (FITDOG) method is used to analyze food polysaccharides by breaking them down into smaller oligosaccharides, which can then be identified and quantified via LC‒MS (Amicucci et al. 2020; Bacalzo et al. 2022; Castillo et al. 2021; Nandita et al. 2021). The FITDOG method for polysaccharide analysis involves several sequential steps for identification and quantification. First, the polysaccharide sample undergoes oxidative cleavage using a Fenton reagent, a mixture of iron (III) sulfate and hydrogen peroxide, which generates reactive oxygen species that cleave glycosidic bonds, breaking the polysaccharide into smaller oligosaccharide fragments. Next, the resulting oligosaccharides are reduced with sodium borohydride (NaBH₄) to stabilize the fragments. Following reduction, the oligosaccharides are separated and purified via solid‐phase extraction (SPE). The purified oligosaccharides are then analyzed via LC‒MS, and identification and quantification are performed by comparing the obtained MS data to those in a reference library of known oligosaccharide fingerprints, allowing the determination of the parent polysaccharide and its relative abundance upon peak area analysis.

This approach offers several considerable advantages for polysaccharide analysis and a powerful and efficient approach to structural elucidation. As a nonenzymatic technique, FITDOG eliminates the need for specific enzymes, making it applicable to a broader range of polysaccharides and allowing for the detailed identification of diverse polysaccharide structures. The generation of characteristic oligosaccharide fragments enables researchers to rapidly reconstruct and predict the complete structure of the parent polysaccharide with high speed and precision. Furthermore, its quantitative capabilities, when utilizing external calibration curves, allow for precise measurement of polysaccharide content (Bacalzo et al. 2022). This method also supports multiplexing, enabling the simultaneous depolymerization of multiple polysaccharides for high‐throughput analysis and further enhancing efficiency due to its compatibility with a 96‐well plate format (Amicucci et al. 2020). By integrating monosaccharide composition, sequencing, and glycosidic linkage determination, this method provides deeper insights into complex carbohydrate systems, thereby advancing glycomics research (Table 1).

### 2.5 MS Analysis of Glycans

In addition to the carbohydrates mentioned above, glycans are also found in various food matrices. Both N‐glycans and O‐glycans are composed of sugar chains attached to glycoproteins but are distinguished by the specific amino acid residue to which they are linked. N‐Glycans are attached to the nitrogen atom on the side chain amide group of Asn residues. All eukaryotic N‐glycans begin with N‐acetylglucosamine (GlcNAc) linked to the Asn residue. O‐Glycans are frequently attached to the oxygen atom in the side chain hydroxyl group of Ser or Thr residues, and the most common type of O‐glycan starts with GalNAc linked to the Ser or Thr residue (Brockhausen et al. 2022; Stanley et al. 2022). Analyzing N‐ and O‐glycans often involves cleaving them from their glycoproteins for MS analysis. To analyze N‐glycans, they are first cleaved from glycoproteins using enzymes called peptide‐N‐glycosidases (PNGases). O‐glycans are typically analyzed by first releasing them from glycoproteins, often using chemical methods like β‐elimination, which can be reductive or nonreductive to preserve the reducing end. The cleaved glycan portions are then analyzed via ESI or MALDI ionization. Isomeric separation is achieved via LC, followed by detection using various MS instruments. This analytical approach closely resembles that used for oligosaccharides (Ruhaak et al. 2018). PGC–MS has become a key technique for glycan analysis due to its superior ability to resolve isomeric structures such as linkage, positional, and anomeric isomers. PGC offers high retention and separation efficiency for both native and permethylated glycans. PGC–MS is compatible with a wide range of glycan classes, including N‐ and O‐glycans, and supports sensitive, high‐throughput profiling in complex biological samples (Ruhaak et al. 2009; Li et al. 2020; Stavenhagen et al. 2015; Zhang et al. 2024).

GSLs are glycolipids, which means that they are lipids attached to a carbohydrate. GSLs are made up of two main parts: a ceramide lipid and a glycan. Ceramide is composed of a sphingosine and a fatty acid linked by an amide bond. The fatty acid can vary widely in length, saturation, and presence of hydroxyl groups, all of which influence the function of the GSL. The carbohydrate portion can vary significantly in terms of its structure and is the basis for GSL classification. These GSLs are classified as neutral GSLs, gangliosides, or sulfatides. MS serves as a powerful tool for GSL analysis, enabling the structural characterization of both the glycan and ceramide components. Common GSL ionization techniques include MALDI and ESI. Additionally, GSLs can be identified separately on the basis of their glycan or ceramide moieties using normal‐phase (NP) and RP HPLC, respectively. High‐resolution MS is utilized for GSL profiling, whereas QqQ MS is applied to quantify known GSLs (Lee et al. 2011, 2012, 2013).

## 3 Comprehensive Glycomic Profiling in Various Food Matrices

MS has become an essential tool for the structural elucidation of carbohydrates, from monosaccharides to complex polysaccharides, offering unparalleled precision and sensitivity. Building upon these advancements, Prof. Carlito B. Lebrilla, a distinguished physical chemist and mass spectrometrist, has revolutionized glycomics research in the fields of food science and nutrition. His pioneering work on MS‐based glycomic profiling laid the groundwork for detailed analyses of complex carbohydrates in diverse food matrices, most notably milk oligosaccharides and dietary fibers. These studies have not only revealed the structural diversity of bioactive carbohydrates but also highlighted their critical roles in promoting gut health and overall human nutrition, marking a transformative shift in glycomics research.

### 3.1 Milk

Milk is the most studied food in terms of glycomics, as it is the ideal source of nutrition for infants and contains essential components for their healthy development. Among the macronutrients from fats, proteins, and carbohydrates, the most abundant component is carbohydrates, which are predominantly composed of lactose units. A unique subset of carbohydrates is HMOs, which are composed of longer oligomers with a lactose core. HMOs are less abundant than lactose but still constitute a large fraction of the dry mass of mother's milk and can even be more abundant than proteins during early lactation (Thurl et al. 2010). In the early 1900s, differences in the fecal composition between breast‐fed and bottle‐fed infants were linked to milk composition. Since the 1930s, the first individual HMOs have been characterized, and extensive studies have been conducted on their roles as growth factors for gut microorganisms, as well as their antiadhesive and anti‐inflammatory properties (Kunz et al. 2014).

Lactose is composed of galactose bound to a reducing glucose residue through a β(1,4) linkage, and HMOs are constructed by the further addition of galactose, glucose, and GlcNAc to the lactose core as well as terminal decorations of fucose and Neu5Ac. Elongated HMOs have either linear or branched structures. The addition of monosaccharide residues can significantly change the chemical characteristics and biological functions of the resulting compounds compared with those of the individual components and lactose. The monosaccharides incorporated determine the charge of the resulting HMO. For example, sialylated HMOs contain a carboxylic acid group, making them anionic. Fucosylated and undecorated HMOs are considered electronically neutral. In addition, adding a monosaccharide with no corresponding glycosyl hydrolases makes the HMO indigestible by the infant, although lactose is readily digestible by human enzymes.

HMOs can consist of 3 to more than 20 monosaccharide units; however, the most prevalent structures exhibit a DP between three and seven (Xu et al. 2017). The potential structural diversity, influenced by variables such as hydroxyl group positioning, α or β configuration, and a branched or linear structure, suggests a wide array of possible structures. Nonetheless, the limited number of glycosyltransferases involved in their biosynthesis results in a defined set of HMO structures present in human milk. While general functions are associated with the entire collection of these structures, individual HMOs exhibit unique and specific biological activities. Therefore, despite the complexity and heterogeneity of HMOs, systematic structural analysis is crucial for understanding the roles of individual molecules.

The Lebrilla group employed various MS techniques to profile diverse HMOs. The neutral and sialylated HMOs were initially fingerprinted with MALDI FTICR MS (Park and Lebrilla 2005). Later, nanoLC separation of the oligosaccharides using microchip‐based columns are employed, and the HMOs were separated on PGC (Ninonuevo et al. 2006; Niñonuevo et al. 2008). Nearly 200 HMO structures have been found in human milk (Ninonuevo et al. 2006). In addition to the PGC stationary phase, the stationary phases HILIC and CE have also been used for HMO separation by other researchers (Auer et al. 2021; Remoroza et al. 2018).

A comprehensive method combining LC‒MS, MS/MS, and sequential exoglycosidase digestion techniques has been developed for the detailed structural elucidation of various HMOs (Wu et al. 2011; Wu et al. 2010). This approach uses LC to separate individual HMO structures, whereas MS provides accurate mass determination. MS/MS, in combination with exoglycosidases, is then employed to identify the monosaccharide components and their specific linkages. Exoglycosidases cleave glycans by selectively targeting terminal residues and are thus highly specific with respect to linkage, stereochemistry, and the configuration of the anomeric carbon at the linkage site. By systematically analyzing each HMO, both the constituent monosaccharides and the precise linkages connecting them were identified.

This comprehensive methodology also allows for the simultaneous monitoring of numerous structures, providing relative quantitation data and making it feasible to conduct large clinical trials with comprehensive HMO analyses (Totten et al. 2014). For example, a study analyzed HMOs in breast milk from 60 Gambian women, revealing significant variations in fucosylation levels on the basis of the secretor and Lewis blood type (Totten et al. 2012). Additionally, a large‐scale study examined over 2000 breast milk samples from 1090 mothers across 15 geographically diverse locations to explore the variation in HMO composition across regions (Vinjamuri et al. 2022). Key insights from these studies reveal that HMO composition varies significantly between individuals and is influenced by factors such as secretor status, Lewis blood type, lactation stage, and geographical location. For example, 2ʹ‐FL serves as a reliable marker of secretor status, and the HMO profile changes over the course of lactation, with the total HMO concentration decreasing while the fucosylation percentage increases. Geographical location also plays a role, with differences in HMO composition observed in various regions (Totten et al. 2012; Vinjamuri et al. 2022; Xu et al. 2017).

In addition to analyzing HMOs in breast milk, MS techniques have been applied to analyze HMOs in other biological samples. HMOs are not digested by the infant and reach their gut. Bacterial consumption studies have shown that HMOs are selectively metabolized by specific gut bacteria, particularly *Bifidobacteria*, through strain‐specific mechanisms (LoCascio et al. 2007; Ward et al. 2006). In particular, *Bifidobacterium longum* subsp. *infantis* (*B. infantis*) is known for its ability to metabolize HMOs. *B. infantis* contains specific solute‐binding proteins that transport the HMOs into the bacteria, where they are broken down by glycosyl hydrolases (Sela et al. 2008; Sela et al. 2012). Correlation analysis of the gut microbiota communities and specific HMO structures in fecal samples from breastfed infants revealed that HMOs influence the composition of the infant's gut microbiota by selectively feeding specific bacteria (De Leoz et al. 2015). In early infancy, the gut microbiota shifts from containing bacteria that do not consume HMOs to containing, for example, *Bacteroidaceae* and *Bifidobacteriaceae*, which do consume HMOs. The presence of HMOs can help *B. infantis* become a dominant bacterium in the infant gut. During early lactation, many of the HMOs provided in milk are lost in feces, but when the bifidobacterial population increases and becomes established, fewer HMOs are found in the feces, indicating their consumption by these bacteria. Glycomic analysis revealed that HMO consumption is highly structure specific, with unique isomers being consumed and others passing through the gut unaltered.

**Figure 2:**
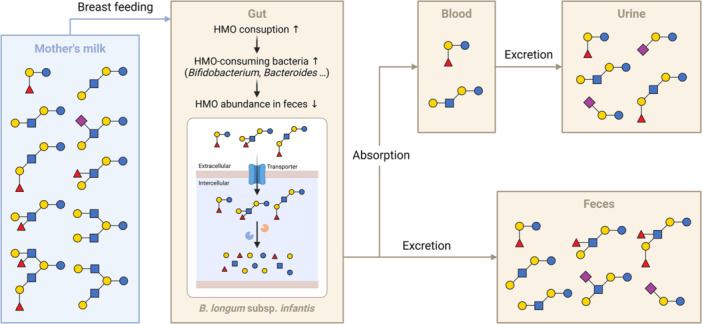
Tracking HMOs in an infant's body from ingestion to excretion. [Color figure can be viewed at wileyonlinelibrary.com]

Furthermore, the composition and quantity of oligosaccharides vary in samples from human milk, feces, and urine, reflecting processes such as selective absorption, intestinal modification, and de novo synthesis (Figure 2). The presence of HMOs in infant plasma was confirmed, demonstrating that HMOs are absorbed in the gut for their subsequent circulation in the bloodstream (Ruhaak et al. 2014). The increased abundance of sialylated oligosaccharides in urine and plasma suggests their potential synthesis in the infant's proximal intestine (De Leoz et al. 2013; Underwood et al. 2015). Such analyses of HMOs in these diverse biological samples provides crucial information on their bioavailability and metabolism in infants.

The composition and diversity of milk oligosaccharides across various mammalian species were also investigated by MS. This analytical approach allowed for the detailed characterization of oligosaccharide, revealing important differences in both structure and function between species. One of the most important comparisons is between human milk and bovine milk. Human milk is known for its exceptionally high concentrations of oligosaccharides, with levels ranging from 20 to 23 g/L in colostrum and 7–12 g/L in mature milk. In contrast, bovine milk contains significantly lower concentrations of oligosaccharides, approximately 20 times less in colostrum and even less in mature milk (Tao et al. 2009). The structural complexity of these oligosaccharides also varies greatly. HMOs are typically larger and more complex than those found in bovine milk. Additionally, although both human and bovine milk oligosaccharides (BMOs) share a lactose core, BMOs also include lactosamine units, which are absent in human milk. Another key distinction lies in the degree of fucosylation. HMOs are highly fucosylated, with over 70% of oligosaccharide structures containing fucose, whereas BMOs show very little to no fucosylation (Aldredge et al. 2013; Tao et al. 2008). On the other hand, bovine milk shows greater sialylation, with nearly 70% of its oligosaccharides containing sialic acid, compared with only 20% in human milk. Additionally, bovine milk contains N‐glycolylneuraminic acid, a form of sialic acid that is completely absent in human milk (Tao et al. 2009). Research on primates has revealed diverse oligosaccharide compositions that are not always aligned with evolutionary phylogeny, suggesting other adaptive factors (Tao et al. 2011). These variations offer insights into mammalian evolution, aid in developing better infant formulas, and hold potential for developing novel biotherapeutics owing to the biological activities of oligosaccharides.

Glycans such as N‐glycans and GSLs in milk can be detected by MS. Human milk N‐glycans are composed of various monosaccharides, including hexoses (mannose, glucose, and galactose), fucose, N‐acetylhexosamines, and sialic acid. These sugars are linked to generate either branched or linear constructs with a core structure composed of two N‐acetylglucosamine and three mannose residues, yielding a wide array of possible N‐glycan structures (Nwosu et al. 2012). Among GSLs, the most abundant milk gangliosides (anionic glycosphingolipids) found were GM3 and GD3. GM3 is composed of a ceramide base, followed by a simple trisaccharide of glucose, galactose, and one sialic acid, whereas GD3 contains an additional sialic acid moiety. MS techniques such as MALDI FTICR MS and HPLC Q‐TOF MS have been used to analyze N‐glycans and gangliosides quantitatively (Lee et al. 2012). UHPLC QqQ MS has also been used for quantitative analysis (Lee et al. 2013).

The analysis of milk oligosaccharides, particularly MS‐based analyses, holds great potential for advancing both scientific understanding and practical applications. Recent research has focused on developing efficient methods to produce key oligosaccharides such as 2ʹ‐FL and 6ʹ‐sialyllactose (6ʹ‐SL) on a large scale, which is critical for their incorporation into infant formulas and other nutritional products (Neu et al. 2024). These oligosaccharides, which are structurally similar to those found in human milk, play essential roles in promoting infant health by supporting immune function and fostering the development of a beneficial microbiome. As research progresses, continued advancements in MS techniques will be crucial in uncovering the full potential of milk oligosaccharides, enabling more accurate structural characterization and facilitating their broader use in health‐related industries.

### 3.2 Food Glycomic Databases

MS is now considered essential because it provides detailed information regarding the complex carbohydrate structures in food, going beyond the limitations of traditional methods that only measure total carbohydrates, fiber, and simple sugars. The Lebrilla group developed a comprehensive monosaccharide database containing more than 800 foods named the Davis Food Glycopedia (DFG) (Castillo et al. 2022). They employed a UHPLC QqQ MS method to analyze 14 monosaccharides across different food categories, including fruits, vegetables, grains, dairy, and processed foods. This method allows the accurate identification and quantitation of individual monosaccharides across a wide array of foods. While pioneering in its own right, this study focused mainly on the monosaccharide compositions of foods without providing detailed structural information.

**Figure 3:**
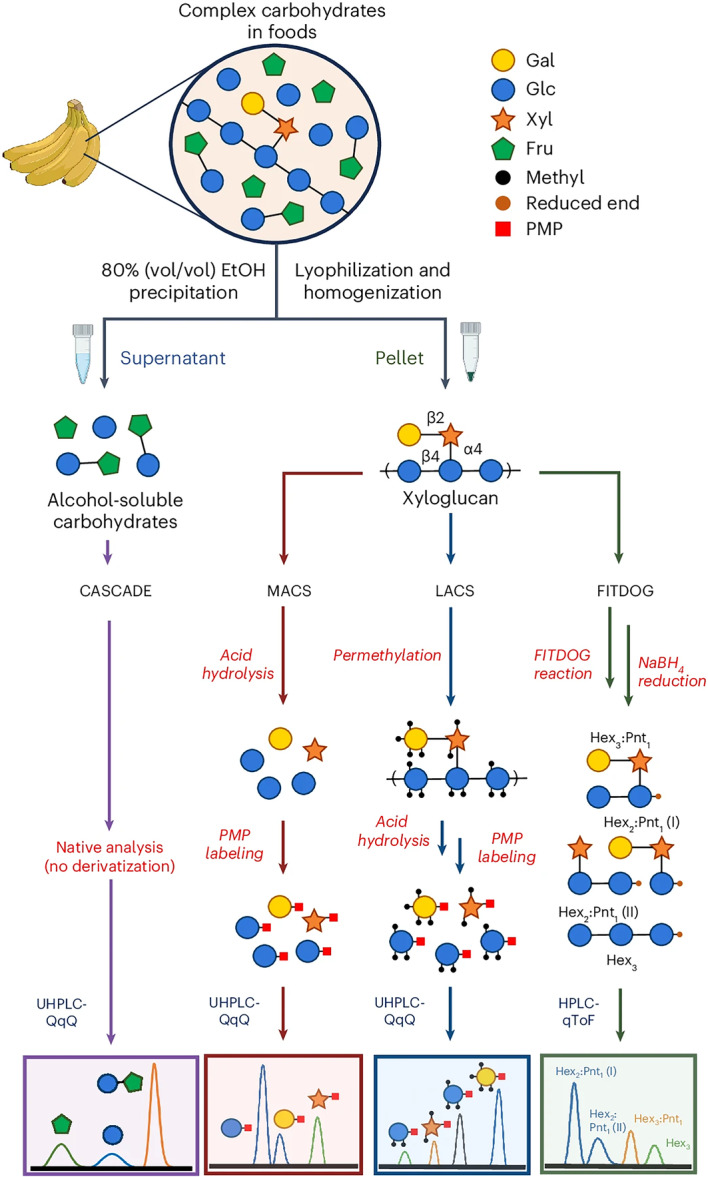
Food carbohydrates quantified and structurally elucidated by CASCADE (far left panel), monomeric analysis of the carbohydrates (left), linkage analysis of the carbohydrates (right), and FITDOG analysis (far right) via rapid‐throughput chemical and LC‒MS/MS methods. Reprinted with permission from Couture et al. (2024). Copyright 2025 (Nature Protocol). [Color figure can be viewed at wileyonlinelibrary.com]

**Figure 4:**
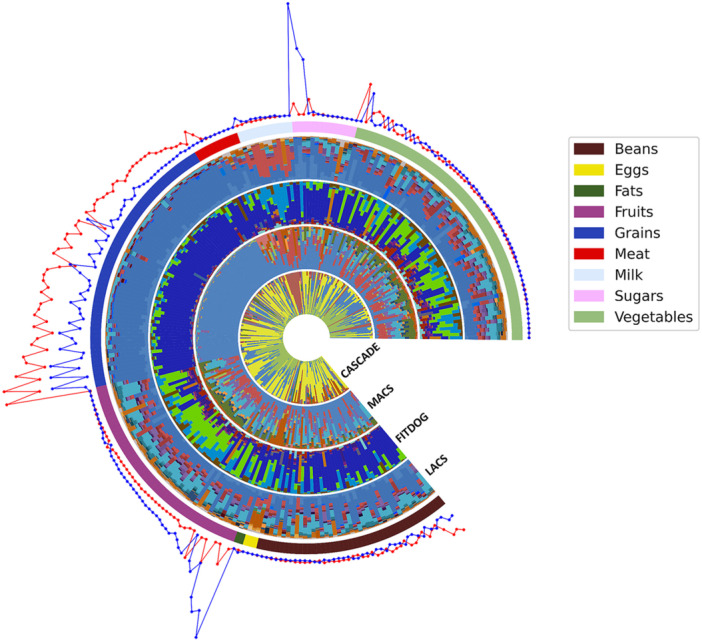
The food carbohydrate database. Relative abundances for all 250 foods were determined for each analysis of the multiglycomic workflow (MACS, LACS, FITDOG, CASCADE) and arranged by food group. The outermost layer represents the absolute quantity of carbohydrates for alcohol insoluble carbohydrates (red) and alcohol soluble carbohydrates (blue). Colors within each ring represent distinct glycan features and a full legend is available in the original article. Reprinted with permission from Suarez et al. (2025). Copyright 2025 (Elsevier). [Color figure can be viewed at wileyonlinelibrary.com]

Recently, a multiglycomic platform that extends beyond only monosaccharide composition was developed (Couture et al. 2024). This platform incorporates several advanced methodologies to characterize food carbohydrates in detail. The monomeric analysis of carbohydrates (MACS) method allows the absolute quantification of monosaccharides. The platform also introduces linkage analysis of carbohydrates (LACS), which provides critical structural insights by identifying glycosidic linkages between monosaccharides, an aspect previously unaddressed. Furthermore, FITDOG is employed to quantify and identify key polysaccharides in food matrices, offering a deeper understanding of their distribution across various foods. Additionally, the platform includes alcohol‐soluble carbohydrate analysis (combined alcohol‐soluble carbohydrate determination; CASCADE), which determines free monosaccharides, disaccharides, and oligosaccharides (Figure 3) (Ehlers Cheang et al. 2024). This multiglycomic approach enables more thorough and precise profiling of carbohydrates in food systems (Figure 4) (Suarez et al. 2025). The Food Glycomic Database serves as a critical resource for advancing carbohydrate characterization, precision nutrition, and diet‒health relationship studies (Jensen et al. 2024; Larke et al. 2023; Suarez et al. 2025). Its ability to map glycan intake from dietary surveys enables more precise nutritional assessments, while its strong predictive power for metabolic outcomes surpasses traditional food composition data. For example, this level of structural resolution enables dietary assessments to move beyond general macronutrient categories such as “total carbohydrates” or “dietary fiber,” which often lack molecular specificity. When integrated with machine learning models, these glycan‐specific data offer improved predictive capacity for metabolic outcomes, with certain glycans, such as raffinose and xyloglucan, outperforming conventional markers like total sugars or fiber in predicting insulin resistance phenotypes (Suarez et al. 2025). In addition, the database can be applied to inform dietary choices, support agricultural advancements in carbohydrate composition, and integrate multi‐glycomic analytical workflows, making it an essential tool for both fundamental research and applied food science.

## 4 Microbial Transformations of Dietary Fibers

As discussed in the previous section, dietary carbohydrates play diverse roles in human nutrition. While some are digested and absorbed in the upper gastrointestinal tract, others—particularly oligosaccharides and dietary fibers—resist enzymatic digestion and pass intact into the colon. There, they serve as substrates for the gut microbiota, which harbors a wide array of carbohydrate‐active enzymes (CAZymes) capable of degrading complex glycans (Flint et al. 2012). The fermentation of these carbohydrates results in the production of SCFAs, such as acetate, propionate, and butyrate. SCFAs are absorbed and metabolized by the host, exerting numerous beneficial effects on human health, including modulation of the immune system and provision of energy.

The composition and activity of the gut microbiota are significantly influenced by the structural complexity of carbohydrates, including their glycosidic linkages and DP (Lee, Song et al. 2024; Ye et al. 2022). Recent work by the Lebrilla and Gordon groups showed that pea and orange fiber intake leads to fiber‐specific alterations in the gut microbiota and host plasma proteome in both gnotobiotic mice and obese adults (Delannoy‐Bruno et al. 2022). Distinct glycosidic linkages in these fibers were selectively degraded by gut microbes, as measured by UHPLC QqQ MS, and correlated with increased expression of CAZyme genes, including glycoside hydrolase (GH) and polysaccharide lyase (PL) genes. Pea fiber enhanced the abundance of enzymes targeting arabinan and galactans, while orange fiber promoted expression of pectate lyases and β‐galactosidases. These microbial responses were aligned with the dominant monosaccharide components of each fiber and were further linked to changes in host metabolic markers. Together, these findings suggest that specific dietary fibers can shape host physiology through microbiota‐mediated glycan metabolism.

**Figure 5:**
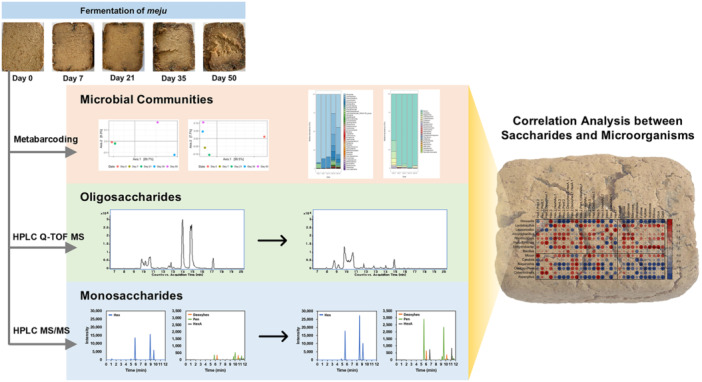
Insights into microbial and carbohydrate changes during fermented food generation through microbial community analysis and advanced glycan analysis via MS. Identification of key microbial players responsible for carbohydrate transformations using an integrated approach. [Color figure can be viewed at wileyonlinelibrary.com]

Beyond the relationship between dietary fiber and gut microbiota, recent research has also explored the dynamic interactions between carbohydrates and fermentative microorganisms in traditional fermented foods. This emerging area has highlighted the transformation of carbohydrate structures during fermentation and the potential generation of novel bioactive glycans. In particular, fermented soybean products have attracted attention due to the formation of structurally diverse oligosaccharides with potential health effects. Advanced mass spectrometry platforms, such as high‐resolution LC‒MS and MS/MS, enable detailed profiling of carbohydrate composition, DP, and structural features, including linkage positions and monosaccharide sequences. Quantitative techniques like HPLC QqQ MS have further improved the accuracy of monosaccharide measurements (Song et al. 2024). In a recent study, Lee, Jo et al. (2024) employed HPLC Q‐TOF MS to identify 19 distinct oligosaccharides in soybean bricks (*meju*), including 9 novel structures likely derived from microbial exopolysaccharides. Similar oligosaccharides with varied degrees of polymerization (DP 3–7) were detected in soybean paste (*doenjang*) and soy sauce (*ganjang*) (Song et al. 2022). While soybeans naturally contain raffinose‐series oligosaccharides (e.g., raffinose, stachyose, and verbascose), these native compounds diminish during fermentation as new glycans emerge. Integrated microbial and glycomic analyses have revealed that *Mucor* initially dominates the fermentation process by degrading soybean dietary fiber, generating oligosaccharides and monosaccharides. *Weissella* subsequently metabolize these carbohydrates, further influencing the structural and functional properties of the fermented product. These microbial successions and their enzymatic activities ultimately shape the glycan composition and functionality of fermented foods (Figure 5) (Lee, Jo et al. 2024). Such findings underscore the utility of MS‐based glycomics in unraveling fermentation‐driven carbohydrate remodeling and guiding the development of functional foods with targeted health benefits.

## 5 Conclusions

In recent decades, MS‐based glycomic tools have made remarkable advancements. Professor Carlito B. Lebrilla, as a physical chemist, has significantly contributed to the development of these tools, applying them to uncover new insights into food science and nutrition. Notably, his work has enabled the precise, high‐throughput structural analysis of complex carbohydrates such as HMOs and dietary fibers, which were previously challenging to analyze with conventional methods. His contributions have laid the groundwork for research on the intricate relationships among dietary carbohydrates and the gut microbiota and health, highlighting the need for robust analytical tools to characterize food carbohydrates effectively.

Despite these advancements, significant knowledge gaps remain, particularly in understanding the precise structure–function relationships of oligosaccharides and their fermentation dynamics in the gut and fermented foods. Further research is essential to address these gaps, refine experimental methodologies, and explore opportunities for precision health applications using functional carbohydrates and microorganisms. Advancing research in these areas will not only lead to the discovery of novel bioactive carbohydrates but also support the development of tailored carbohydrate‐rich foods designed to meet individual health needs.

## Author Contributions

**JaeHui Song:** writing – original draft, investigation, conceptualization, data curation, methodology. **HyunJi Lee:** resources, investigation, writing – original draft, visualization. **Youngshik Choe:** software, supervision, resources, visualization, funding acquisition, writing – review and editing. **Hyeyoung Lee:** writing – review and editing, conceptualization, funding acquisition, visualization, formal analysis, project administration, supervision, validation.
